# Optimizing Health Across Humans, Animals, Plants, and Ecosystems: How Long Before Benefits Turn Harmful—and Harm Becomes Healing?

**DOI:** 10.1093/ofid/ofaf310

**Published:** 2025-10-10

**Authors:** Patrick Giraudoux, Dominique Bourg, Thierry Lefrançois, Didier Bompangue, Dominique Angèle Vuitton, Denis Malvy

**Affiliations:** Chrono-environment, Université Marie et Louis Pasteur/Centre National de la Recherche Scientifique, Besançon, France; Faculté de Géosciences et de l'Environnement, Université de Lausanne, Switzerland; Institut Supérieur de Philosophie, Université Catholique de Louvain, Louvain, Belgium; Moroccan Royal Academy, Rabat, Marocco; Direction Générale, Centre de coopération internationale en recherche agronomique pour le développement, Paris, France; Institut One Health pour l'Afrique, Université de Kinshasa, Kinshasa, Democratic Republic of the Congo; Chrono-environment, Université Marie et Louis Pasteur/Centre National de la Recherche Scientifique, Besançon, France; Université Marie et Louis Pasteur, Besançon, France; Global Health in the Global South Research Team, National Institute for Health and Medical Research Unité mixte de recherche 1219 and French Research Institute for Sustainable Development Equipe mixte de recherche 271, Bordeaux Population Health Research Centre, University of Bordeaux, Bordeaux, France; Department of Infectious Diseases and Tropical Medicine, Division of Medical Specialities, Bordeaux University Hospital Centre, Bordeaux, France

**Keywords:** anthropocentrism, biodiversity, ethics, nature value, One Health

## Abstract

The One Health framework endorsed by the quadripartite (World Health Organization, World Organisation for Animal Health, Food and Agriculture Organization, and United Nations Environment Program) is defined, in part, as an “integrated, unifying approach that aims to sustainably balance and optimize the health of people, animals, and ecosystems,” and it explicitly refers to the health of plants as part of the whole interrelated system to consider. Although the ultimate issue is the planet's habitability for humans, the definition introduces a shift in perspective—human health is no longer the sole priority but must be balanced and optimized alongside the health of animals, plants, and ecosystems. This raises some practical and ethical questions. Drawing on case studies and the framework of the IPBES (Intergovernmental Science-Policy Platform on Biodiversity and Ecosystem Services) on the values of nature, this study explores the implications of a global approach to health, with the ethical and practical questions that it raises.

## Teaser

The One Health definition—endorsed by the World Health Organization, World Organisation for Animal Health, Food and Agriculture Organization, and United Nations Environment Program—raises some practical and ethical questions that are explored through case studies and nature grid values per the Intergovernmental Science-Policy Platform on Biodiversity and Ecosystem Services.

The World Health Organization constitution defines human health as “a state of complete physical, mental and social well-being and not merely the absence of disease or infirmity” [1]. The World Organisation for Animal Health provides no explicit definition of animal health other than describing it as a key component of animal welfare, which is defined according to the 5 “freedoms”: from hunger, malnutrition, and thirst; from fear and distress; from heat stress or physical discomfort; from pain, injury, and disease; and to express normal patterns of behavior [2]. Although the Food and Agriculture Organization (FAO) organizes an International Day of Plant Health, a specific definition of plant health remains unestablished. In addition, ecosystem health remains undefined by the FAO and the United Nations Environment Program, with the literature reflecting a lack of consensus on its meaning. Giraudoux [3] proposed that “an ecosystem is healthy if it preserves the maximum potential of its functions, dynamics and evolutionary capacities, as well as those of the systems it includes, and which include it.”

However, despite the lack of a fully satisfactory definition for each component of One Health—as defined by the One Health High-Level Expert Panel and jointly endorsed by the quadripartite World Health Organization, World Organisation for Animal Health, FAO, and United Nations Environment Program [4]—the concept remains operational in practice. Evidence-based practices are already being implemented by medical doctors, veterinarians, agronomists, and ecologic engineers who diagnose and propose ways to restore and maintain the health of living systems within their domains of responsibility. These practices are generally implemented separately without considering the consequences of specific decisions on any other form of health.

The concept of sustainable health balance and optimization across humans, animals, plants, and ecosystems implies a system-wide approach to health rather than a component-specific one. In this context, human health may be intentionally constrained to preserve the health of animals, plants, or ecosystems. For example, marshes may be protected despite serving as malaria sources due to their essential role in water regulation and the support of unique biodiversity. This raises ethical issues to develop normative and legal aspects with the question of personal rights for animals and the legal status of ecosystems [5]. Beyond the technical need for an integrative approach to decision making across administrations and private sectors accustomed to working in silos, this shift demands a cultural transformation among health actors within each of these sectors. This integrated perspective on health is likely difficult for medical doctors and the broader society to accept, as they have been trained in or become accustomed to treating human diseases at any cost. The prevailing mindset often focuses on eliminating or even eradicating human diseases with little or sometimes no concern for the unintended consequences on animals, plants, and ecosystems. In addition, prevention is often reactive, implemented after people become ill, despite clear evidence that managing environmental components (eg, wildlife, habitats, and biodiversity) and human behavior is essential to preventing disease occurrence [6].

**Figure 1.**
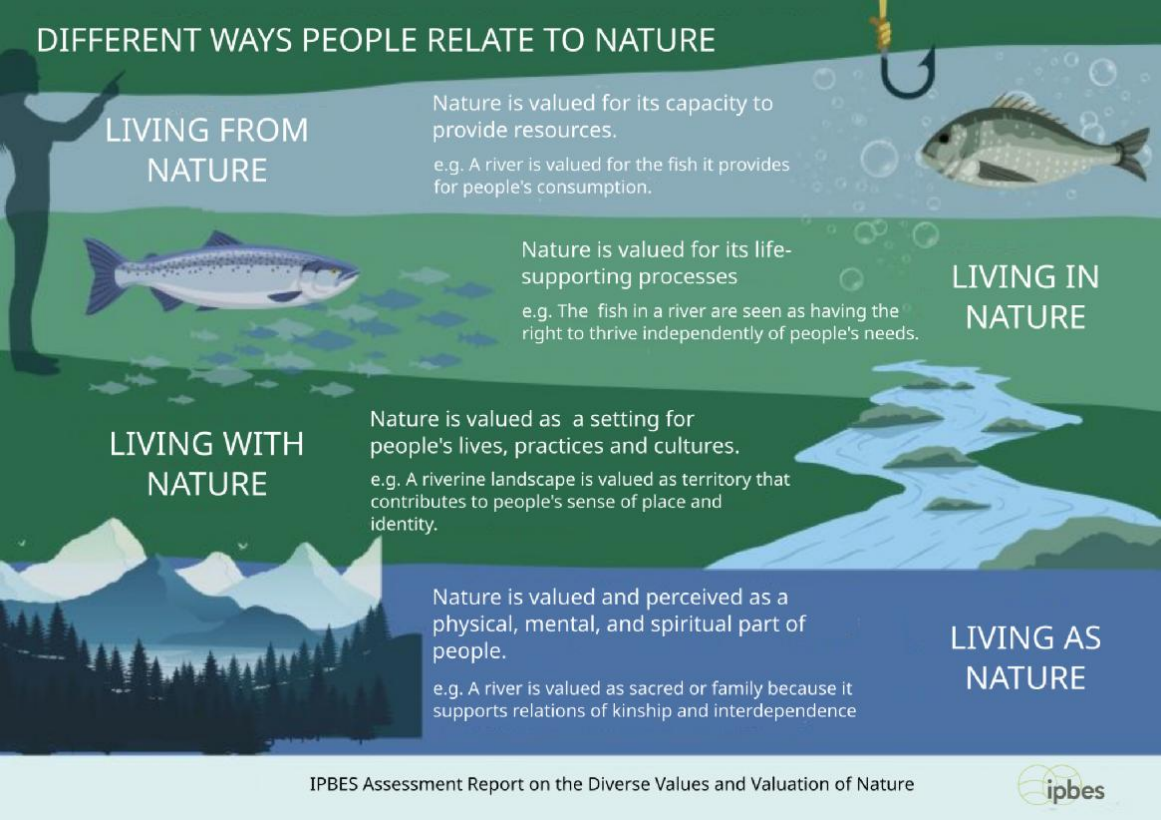
Different ways that people relate to nature after Anderson et al [12].

The origin of this low level of concern for nonhumans lies in our identity as “moderns” [7, 8]—that is, as heirs to a worldview shaped by the advent of classical physics and reinforced by certain interpretations of Genesis 1:26–28, which gained prominence in the late Middle Ages and depict humans as uniquely created in the image of God. This perspective, further supported by Descartes’s philosophy, has led us to perceive ourselves as separate from nature and its ecosystems [9–11]. Therefore, human health has been conceived as detached from ecosystems and other species, which are destined to be eradicated if they cause disease. In this study, we propose to examine what is inferred, on the contrary, by the notion of One Health “balancing and optimizing the health of people, animals, plants, and ecosystems” within the framework proposed by the Intergovernmental Science-Policy Platform on Biodiversity and Ecosystem Services about the values of nature [12]. Four types of relationships with nature have been proposed: living “from,” “with,” “in,” or “as” nature. Here, the term “nature” can be understood in the first 3 cases as synonymous with “nonhuman” and in the last one as “the whole of the world” [7, 13]. We can live from nature: from the various resources we draw from it. We can live with nature: we rub shoulders with life beings other than humans, however endowed with their own interests. We can live in nature: a living environment, landscapes with which we can, to varying degrees, identify ourselves. Finally, we can live as nature: that is, as integral parts of a whole that transcends us—the question of the intrinsic value of every element of nature here is at stake (Figure 1).

The hallmark of *modernity* has been to reduce these 4 modalities to “living from” nature as a resource, conceived as natural capital devoid of intrinsic value and destined to be transformed into economic goods and ecosystem services (for the single benefits of humans) [14]. Therefore, in the countryside and cities, “living with” must be reduced to a minimum. “Living in” has been preserved only in a recreational sense, at best. As for “living as,” this is vehemently denied in the name of a principled strangeness of humanity to nature.

In this study, through case studies and assisted by an environmental philosopher (D. B.) [15], we present the views and questions of 5 field actors: 3 medical doctors (D. M., D. B., and D. A. V.), a veterinarian (T. L.), and an ecologist (P. G.). All have implemented the One Health approach in their fields of expertise for the past 3 decades [16–20] and fully adhere to the new definition of One Health. We argue that the One Health framework, which emphasizes that the health of humans, animals, plants, and ecosystems must be “balanced and optimized,” requires more than an expanded systems approach to the concept of health. It also raises ethical issues that are now unresolved [5, 21].

## EBOLA: CONSEQUENCES OF JUST “LIVING FROM”

The Ebola virus is a zoonotic agent responsible for severe human disease and recent large outbreaks of hemorrhagic fever in Central and West Africa. Despite limited understanding of animal reservoir ecology, the current consensus holds that human infection originates from contact with infected bushmeat, including bats, great apes, and possibly other mammals [19]. Moreover, Ebola disease can cause massive die-offs in great ape populations, such as gorillas and chimpanzees, and subsequent conservation problems [22]. Factors contributing to the emergence of recent Ebola virus disease epidemics in humans have been linked to the opportunistic encroachment of humans into the forest habitats of wildlife species [23], with the increased exploitation of remote forest areas, changes to the agricultural destination of forest ecosystems [24], and the extensive spatial and technological connectivity of associated populations [25, 26]. These factors often suggest an increased risk of emerging infectious diseases in West and Central Africa caused by known and unknown pathogens and involving known and unknown reservoir and intermediate hosts [27]. In the early Ebola outbreaks, the initial zoonotic introduction from a wildlife reservoir in a remote and difficult-to-access densely forested area systematically led to limited human-to-human transmission and rapid containment, contrary to the following epidemic crises. The regions where this disease is emerging or reemerging are primarily areas facing notable challenges, marked by geopolitical volatility, recurrent internal armed conflicts [28], and substantial cross-border movements with neighboring countries, reflecting, among other things, civil or socioeconomic insecurity [29].

Strikingly, notable lifestyle shifts have occurred among autochthonous populations in regions affected by emerging and reemerging pathogens in the past decades. In the Democratic Republic of Congo, these changes affect 2 ethnic groups: the Bantus and the autochthonous communities formerly known as the Pygmies. Pygmies have always lived in the forest, hunting and gathering. Trading for wealth was not part of their way of life. In the event of an unexplained death, the camp was burned, and the small community relocated to a new site. Over time, modernization programs targeting indigenous communities have led to resettlement. For those not relegated to low-level employment or begging, traditional lifeways have shifted toward income generation, primarily through hunting, now practiced not only for subsistence but increasingly and predominantly for trade [30]. Similar changes are occurring among forest-dwelling Bantu communities, marked by the dismantling of ancestral codes that once structured their traditional lifeways and regulated hunting practices. For example, the rules laid down in these codes may specify prohibited or regulated areas of access, hunting periods and methods, and the profile of animals to be hunted, banning the accidental collection of animal corpses or the hunting of weakened prey.

Anthropogenic intrusions into forest ecosystems driven by surrounding human activities promote wildlife hunting, creating a relatively profitable bushmeat trade that is widely accessible and sold in local markets [27, 31]. The accumulation of economic and political crises, demographic pressure with migratory flows mixing urban and rural populations, and the opportunity of a food demand market for meat products have contributed to the fragmentation of the social fabric of these peoples and their tried-and-tested relationship with nature and the forest environment.

### Take-home Message/Questions

A joint upheaval of communication and economic structures—and therefore of ancestral rules adapted to the avoidance of epidemics—may have led to the increasing emergence of some infectious diseases. Therefore, a One Health approach should promote the development of new human-nonhuman relationship frameworks that support biodiversity conservation and human health preservation.

The changing face of Ebola illustrates the near-universal spread of the Western economic model of human-nature relations and the resulting impasses that it creates. Strikingly, the Ebola case study evokes a so-called tragedy of the commons [32, 33] where forest ecosystems are overexploited and destroyed. In sum, this model recognizes only the “living from,” the economically oriented extraction of resources, and denies the other 3 modalities mentioned. Only human beings and nonhuman “things” are recognized, with rule making reserved exclusively for human-human relations. Conversely, recognizing that we coexist with other living beings, including microbes, requires the regulation of human-nonhuman relationships. These were the functions of the, generally nonwritten but strictly followed, various prohibitions described so far. New rules must be devised to govern human-nonhuman relations, driven in this case by our own interests.

## THE WORLDWIDE SPREAD OF ATOPIC DISORDERS: THE NECESSITY OF “LIVING WITH”

Atopic disorders such as atopic dermatitis, hay fever/allergic rhinitis, and some forms of asthma underwent a steady increase in the second half of the 20th century, with growing prevalence rates from 5% to 30% and more so in high- and middle-income countries. This trend is a unique phenomenon in the history of humankind and is strikingly associated with the increase in the urban way of life of the worldwide population [34]. The marked increase in autoimmune diseases and chronic inflammatory disorders is also concerning and shares similar dysfunctions of the immune system. Well-established risk factors include living in urban areas, residing in regions with low ultraviolet light, consuming a Western diet, and repeated antibiotic exposure early in life. Changes in the exposome specifically related to urban life (eg, pollution gas, microparticles from various origins, new allergens introduced in the cities) have been postulated to play a role in the rising atopic allergic diseases and the severity of those related to respiratory health. Most research funding is dedicated to studying such “city-related risk factors.” Conversely, environmental epidemiology findings may support the “hygiene hypothesis,” which suggests that reduced or altered early-life microbial exposure due to environmental and hygiene changes leads to improper immune system development and increases the likelihood of allergic and inflammatory diseases. The increase in atopic allergic diseases would thus be the consequence of the recent loss of a style of life established from the beginning of humanity.

Numerous cross-sectional studies, comprehensive longitudinal cohort studies of children living in various environment types, and experimental studies have provided scientific support for a concept derived from the hygiene hypothesis, which stresses the immunologically mediated “protective” potential of microbial and antigenic biodiversity. For instance, in the PASTURE project, the authors report the results of a longitudinal cohort of 1000 Austrian, Finnish, French, German, and Swiss children established in 2001 and followed up to date. Its main aim was to compare children exposed or not exposed to the traditional dairy farm environment [35, 36]. The study results supported the independent protective role of traditional farm life in childhood against the occurrence of various allergic diseases and respiratory infections later. This protective role was linked to exposure to a high diversity of animals, microbial agents, and antigens associated with traditional farming lifestyles. The protective effect was even more pronounced when these farms were grouped in hamlets or villages. Residential proximity to a manure facility was also identified as an independent protective factor. Similar findings have emerged from observational studies comparing Amish and Hutterite families in the United States, both of which descend from common European ancestors. The Amish have preserved traditional farming practices, including the regular presence of mothers and children in stables and barns. In contrast, the Hutterites have adopted highly mechanized agricultural methods, with no maternal involvement in farm activities, and their children exhibit an asthma prevalence >6 times higher [37]. Furthermore, the PASTURE study and the complementary GABRIELA project, involving >8000 children, demonstrated a strong and independent inverse association between raw milk consumption—maternal during pregnancy and pediatric after weaning—and clinical indicators of allergic manifestations. Despite a considerable decrease in the total number of microorganisms in raw milk over the past 20 years, the diversity of living microbial species remains a major distinction from heat-treated milk. This microbial diversity, with marked differences in protein and lipid composition, such as omega-3 and short-chain fatty acids, may also influence gut microbiota. Further studies showed that the asthma-protective effect of farms was associated with a richer home dust microbiota composition [38], suggesting an immunoregulatory role on the gut microbiota and the dependence of this relationship on the environment, including the maternal environment during pregnancy [39].

However, recommending frequent contact with farm animals and the consumption of raw milk or raw milk dairy products presents a dilemma for public health actors: hygiene and pasteurization are historically linked to progress, modernity, and the reduction of infectious diseases such as tuberculosis and brucellosis. Considerable progress had been made in eliminating major pathogens historically responsible for these severe diseases through the control of farm animal health and the sanitation of dairy production processes. All studies conducted since 2000 on the influence of the farm environment and raw milk consumption acknowledge this progress and do not challenge its validity. Yet, a few pathogens are still of concern, such as *Listeria* spp for pregnant women and Shiga toxin–producing *Escherichia coli* species for children <5 years of age. Potentially fatal infectious accidents resulting from consuming unpasteurized milk and milk products and/or visiting dairy farms by children are rare in countries with high levels of hygiene [40]. Nevertheless, the medical community generally does not support the beneficial effects of early exposure to diverse nonpathogenic microorganisms, and the benefit-risk ratio is seldom assessed objectively.

### Take-home Message/Questions

The maturation of the human immune system, mostly via gut microbiota, is dependent on the diversity of microorganisms and antigens in the environment. Consequently, excessive hygiene and oversimplified/low-biodiversity (eg, urban) environments have increased atopic disorders and other chronic inflammatory diseases in high- and middle-income countries, with public health consequences in terms of morbidity and mortality. This raises questions about the balance between individual and community benefits. In summary, should we promote healthy ecosystems rich in biodiversity, accepting the risk of infection of a few individuals vulnerable to pathogens for the health benefit of up to a third of the population? The balance between rare cases of acute severe/deadly infections and an estimated 262.41 million prevalent asthma cases globally, with a number of disability-adjusted life years of 21.55 million [41], cannot be eliminated from the arguments supporting public health decisions, at least in countries with an already high level of hygiene and food safety control, which considerably reduce the rate of severe infections.

This case, disregarding the dimension of resource use (the “living from”), highlights the health effects of the denial of “living with.” The desire to completely isolate humanity from contact with other species, including nonpathogenic microorganisms, is detrimental to our own health.

## SCHISTOSOMIASES IN SENEGAL: AN ATTEMPT TO “LIVE IN,” WITH WIN-WIN INTERACTIONS

Schistosomiasis is caused by snail-transmitted flatworms (*Schistosoma* spp) that penetrate the human skin, affecting >250 million people worldwide in 2021 [42]. Control efforts are endless when infections are treated with drugs because people can quickly get reinfected upon returning to snail-infested waters. Furthermore, molluscicides have harmful effects on aquatic biodiversity in treated water bodies and are even banned in freshwater environments in certain regions (eg, Europe). In the St Louis–Richard Toll region of Senegal, the Diama dam was constructed (1981–1986) to increase irrigated agriculture. As a result, the region has seen a notable increase in fertilizer utilization, resulting in the proliferation of underwater vegetation and the provision of habitat and food for snails. Rohr et al [43] implemented a One Health approach, referred to as “planetary health innovation,” that applied multidisciplinary strategies to identify sustainable solutions balancing human and ecosystem health. By targeting socioecosystem components not directly related to health, they demonstrated that exposure and risk could be sustainably reduced. The removal of aquatic vegetation at water points reduced the incidence of schistosomiasis by 30%, creating other win-win points of improvement. The harvested vegetation produces compost that is several dozen times cheaper than synthetic mineral fertilizers and animal feed. Increased production of onion and pepper was observed, and the onion rot was markedly lower in compost-alone plots. This agricultural recovery makes it profitable—and therefore sustainable in this case—to remove vegetation from water points, making them more accessible and healthier without damaging biodiversity and water quality.

### Take-home Message/Questions

Unlike the classic siloed human health approach of using drugs to reduce infection targeted at humans (praziquantel) or intermediate hosts (molluscicides), a system approach involving health professionals, agronomists, and ecologists has led to solutions that “balance human, plant, and ecosystem health,” with win-win results at several points in the socioecosystem. However, this approach alone could not completely eliminate schistosomiasis.

The case of schistosomiases illustrates another denial of the “living in”: the reality that humans not only coexist with other living beings but also inhabit ecosystems composed of multiple species. This embedded proximity can, at times, become unintentionally hazardous. This case is all the more interesting because it shows an effective health strategy built on consideration of the ecosystem and the effects of our actions on it in search of cobenefits. Another crucial point is that once a global, ecosystem-based health strategy is adopted, the goal can no longer be the maximization or perfection of human health but rather its optimization. Such a One Health approach reduces but does not eliminate schistosomiases. One Health can also be conceptualized as a form of community health, which considers the health of other species and the ecosystem, even if it tends to prioritize human health. This principle of nonabsoluteness fundamentally conflicts with the traditional medical approach.

## BARGY IBEX: “LIVING AS” AND THE INTRINSIC VALUE OF LIFE

Brucellosis is a zoonosis caused by the bacteria *Brucella melitensis* and has major economic and public health implications. France has been officially free of bovine brucellosis since 2005. In 2012, when the infection reappeared on a cattle farm in the Bargy mountain range (in the Borne massif of the Alps) and in 2 humans (after consumption of Reblochon cheese made with raw milk), the initiated investigation uncovered infected and seropositive Alpine ibex (*Capra ibex*)—an acting reservoir for brucellosis [44, 45].

In 2012, the French authorities implemented several management strategies, starting with extensive or selective culling operations and capture, test, and removal [46]. This led to a major decrease in brucellosis seroprevalence in ibex (from 50% in 2012% to 4.9% in 2022 in females of the core area) and a decrease in the ibex population (from 570 in 2013 to 270 in 2016 and around 380 in 2022) [47, 48].

Following a new cattle infection case in 2021, the French Ministry of Agriculture aimed to control the disease by reducing ibex prevalence, seeking to lower the risk of transmission to livestock while allowing the epidemic to subside naturally without further compromising ibex conservation. Through modeling, ANSES (Agence nationale de sécurité sanitaire de l’alimentation, de l’environnement et du travail) [48] identified the 2 most favorable scenarios involving small-scale selective culling and the “capture, test, and remove” strategy, considering that eradicating the disease through extensive culling would not guarantee the natural extinction of the disease by 2030. Nevertheless, the local authorities decided to implement this strategy in 2022, with a mass cull of up to 170 ibex, despite 88% opposition in a public consultation and negative advice from the National Council for Nature Protection. A Senate report backed the local authorities’ decision, citing the need to “protect animal and human health” [49].

Such strategies raised ethical and conservation issues, especially as ibex populations have recovered in the Alps in recent decades and the species is now protected in France. Conservation groups took the prefectural decree to court, and the ruling in 2023, after partial implementation of the strategy, overturned the decree.

This example illustrates the competing priorities of the different actors involved. Farmers prioritize the protection of their livestock, knowing that the detection of a positive animal will lead to the culling of the entire herd. Farmers and local authorities prioritize the economic consequences for the Reblochon cheese industry. The Senate prioritizes the economic consequences at the local and national levels; new cases can jeopardize the infection-free status of the country. Hunters favor a massive cull, given the possibility of reintroducing healthy animals from other regions. Conservation organizations prioritize the conservation of ibex, and nature users such as hikers want to preserve the ibex population, especially as these animals are easy to see.

### Take-home Message/Questions

Prioritizing domestic animal protection over wildlife—even in contexts of limited risk to livestock and humans and in contradiction to scientific recommendations—underscores the primacy of perceived economic value over conservation priorities. It ignores several ethical principles related to conservation. The conservation of species diversity has intrinsic, aesthetic, spiritual, and economic values and preserves ecologic complexity and coevolution [50]. Decisions are also driven by the prioritization of human health over not only the health but, at times, very survival of other species. We could argue that a different choice could have been made if the concept of One Health in social-ecologic systems had been implemented at a territorial level (see, eg, Giraudoux [51]). This concept combines and equally values the conservation of natural resources and the health of all species [5].

Similar questions can be raised about insect vectors of infectious diseases, such as various species of mosquitoes that transmit arboviruses (responsible for dengue, West Nile fever, etc) and the tsetse flies, which transmit protozoa responsible for sleeping sickness and African animal trypanosomiasis. These insect vectors have recently become the target of elimination/suppression strategies [52, 53] or even a pan-African eradication campaign [21] to prevent their negative impact on animal and human health. Are insects or protozoan parasites less valuable than wild mammals such as ibex? Attributing intrinsic value to mosquitoes, tsetse flies, or protozoa implies that species, even entire populations, can no longer be regarded solely as instrumental to human ends.

Intrinsic value is often interpreted as implying the attribution of legal personality to these species—that is, recognition as living entities with corresponding rights and duties. This legal recognition of environmental personhood for nonhumans exists in various forms—for example, for ecosystems such as the Whanganui River in New Zealand, the Gange and the Yamuna River in India, and the Mar Menor lagoon in Spain, as well as other rivers and watersheds of Colombia, Bolivia, Ecuador, and the United States. Regarding vectors, this perspective does not preclude population control but rather emphasizes the need to avoid unnecessary harm to the species or its constituent elements, such as populations, and importantly to prevent the extinction of species, subspecies, or local populations.

The intrinsic value and personhood granted to nonhumans imply that any living form should be protected, which includes populations, species, and ecosystems. However, as Bouyer et al [21] outlined, we must kill plants and animals to eat, and eat to live, and it seems unreasonable that our basic biological needs should be morally objectionable. Hence, the protection of individuals cannot be absolute (see, eg, Morizot [54]). Trade-offs among multiple intrinsically valuable living entities imply that harm may be done to protect higher interests or promote the common good. This raises the question of what constitutes necessary harm and the common good in ecosystems, where all entities are interdependent.

This brings us directly to the problem of norms and rules in our relationships with other living beings. We live within ecosystems and within the Earth system, many aspects of which remain poorly understood. In this sense, we are part of a greater whole that transcends us, belonging to a community of living beings without which our own survival would not be possible. We live as nature, as nature itself. This is why other species within a given biotic community can be recognized as possessing intrinsic value. This moral value can give rise to the form of positive law known as the rights of nature. The moral background here is the hierarchical ecocentrism dear to Aldo Leopold [55] and further theorized by John Baird Callicott [56]. While recognizing the intrinsic value of the ibex or the tsetse fly, the strategy described here, when prioritizing human health, focuses on local control of the ungulate or the fly without aiming to eradicate the species.

## CONCLUSION

One Health remains an anthropocentric framework, as its ultimate aim is protecting humans by preserving the long-term habitability of the Earth for them [57]: first, by protecting the various components of ecosystems and their health, without which human life is simply no longer possible; second, by prioritizing our duties toward other living beings and giving priority to human beings over other individuals while recognizing the right to life of their species. The logic at work differs markedly from the logic of modern action, which is a logic of endless siloed goals, such as the accumulation of capital, the quest for a form of “absolute” human health, and the utopian eradication of any kind of difficulty that would impair the survival of humans.

**Figure 2.**
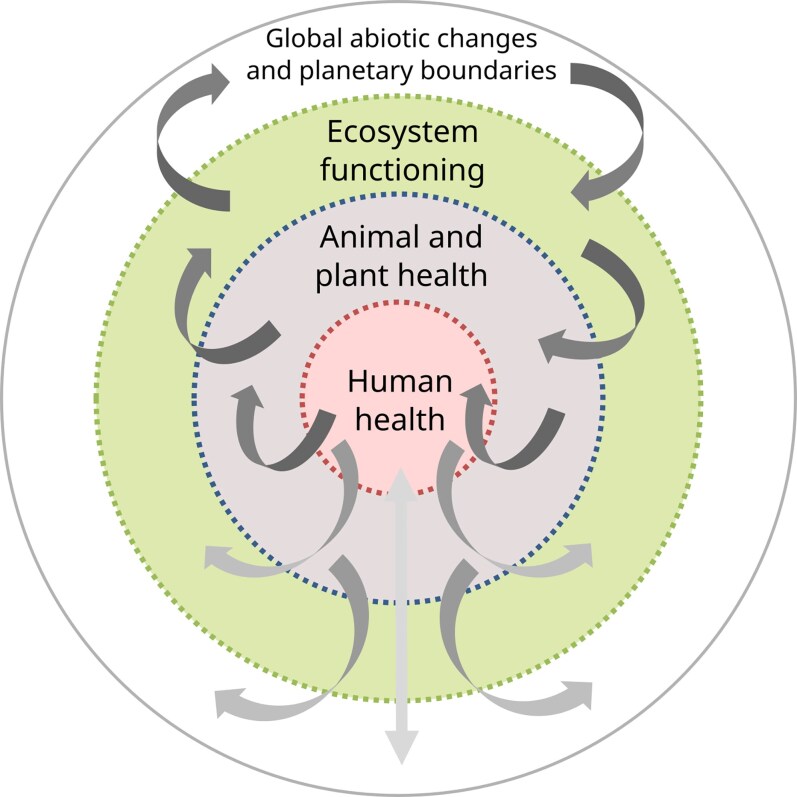
Representation of the hierarchy of constraints on different health types. Human health is seen here as included in and the result of the health of other living organisms within planetary limits. Conversely, the human footprint, linked to the combination of human demography and consumption, is now exerting strong pressure on the systems that determine human health.

In the context of One Health, the aforementioned examples show how good for one target can turn bad for others. All these examples underline “the essential link between human, domestic animal and wildlife health and the threat disease poses to people, their food supplies and economies, and the biodiversity essential to maintaining the healthy environments and functioning ecosystems we all require,” as described in the Manhattan principles, which are at the root of the One Health concept [58]. It also underscores why the management of such complex systems must “mobilize multiple sectors, disciplines, and communities at varying levels of society to work together” [4] to anticipate and prevent the undesirable consequences of decisions made about one element of the One Health triptych if the others are disregarded. “Sustainably balance and optimize the health of people, animals, and ecosystems” [4] is not just a matter for medical doctors and veterinarians but for a much wider part of society, with unavoidable trade-offs among different health outcomes to be considered over time (see Supplementary Appendix 1: the ultimate effects of rabies elimination in Western Europe). This should prompt consideration of human, animal, and ecosystem health not only in terms of their interdependencies but also in their hierarchical organization within a cascade of constraints and feedback mechanisms (Figure 2). This raises the question of what form of governance can ensure effective coordination among all stakeholders and how nonhuman entities can be represented within such a deliberative framework. To the best of our knowledge, ways of addressing the eminent normative and ethical dilemma of the One Health definition of the One Health High-Level Expert Panel remain unestablished. The recent Nexus and Transformative Change assessments of the Intergovernmental Science-Policy Platform on Biodiversity and Ecosystem Services [59, 60] emphasized that the best way to bridge single-issue silos is through integrated and adaptive decision making, called “Nexus approaches.” The One Health concept is undoubtedly a special case of a Nexus approach, but relevant governance organizations capable of addressing such issues, including ethical aspects, need to be invented.

## Supplementary Material

ofaf310_Supplementary_Data

## Supplementary Data

Supplementary materials are available at *Open Forum Infectious Diseases* online. Consisting of data provided by the authors to benefit the reader, the posted materials are not copyedited and are the sole responsibility of the authors, so questions or comments should be addressed to the corresponding author.

## Notes

***Financial support.*** Publication fee supported by the Institut National de la Santé et de la Recherche Médicale.

***Author contributions.*** D. M. and P. G. initiated the project and conceived the topic. P. G. coordinated the writing. All authors provided a case study or philosophical framework and reviewed and discussed successive versions of the article.
