# Supplementary material for: Optimizing Health Across Humans, Animals, Plants, and Ecosystems: How Long Before Benefits Turn Harmful—and Harm Becomes Healing?
Source: Open Forum Infect Dis. 2025 Oct 10;12(10):ofaf310. doi: 10.1093/ofid/ofaf310 (PMC12548796; doi:10.1093/ofid/ofaf310)
Supplement: ofaf310_Supplementary_Data [file ofaf310_supplementary_data.docx]

Supplementary Appendix1: **The ultimate effects of rabies elimination in Western Europe**

In the second half of the 20th century, red fox (*Vulpes vulpes*) populations experienced a rabies epidemic throughout continental Europe. The first cases of rabies in wildlife (foxes and wolves) were observed in 1951 in three districts of eastern Latvia (Wandeler, 2004), and the disease spread south-west toward Poland, with the front of the epizootic then moving about 20–60 km per year. The first cases were reported in Germany in 1947, Belgium and Luxembourg in 1966, Switzerland in 1967, France in 1968, and the Netherlands in 1974 (Delcourt et al., 2022). Hunting before the outbreak (fox pelts were valuable at the time), the outbreak itself, and its management kept fox populations relatively low throughout Europe until the early 1980s. Although no fatal autochthonous human cases were reported during the more than 30 years epidemic due to the lower infectivity of the virus strain to non-fox species, efficient prevention campaigns, and human post-exposure treatment, a response in all these countries was to try to reduce the fox population further (shooting, trapping, poisoning, and gassing), but this failed to stop the epizootic and actually accelerated its spread on the western front. To reduce public health risks to humans and domestic animals, oral fox rabies vaccines were developed. From the mid-1980s, large-scale fox vaccination campaigns were conducted to successfully eliminate fox rabies in the area. The last case of fox rabies was recorded in 1998 in France, 2001 in Germany, and in 2018, fox rabies was officially considered eliminated from most of the EU territory. The front is now on the eastern border of the EU, from Estonia to Romania.

The vaccination campaigns were followed by a demographic explosion, and the fox populations became much larger than before the outbreak. Rabies vaccination alone was not the direct cause of this demographic explosion, as it also occurred in rabies-free areas decades earlier, like in the UK. The causes are more likely to be a combination of human-induced environmental changes that could benefit the continental fox population once rabies has been eliminated and the subsequent systematic and massive culling of foxes has ended (Delcourt et al., 2022). This demographic explosion was followed by the emergence of urban fox populations throughout Europe. Rural fox populations now seem to have reached densities close to carrying capacity and populations stabilized, but they fluctuate locally due to local epizootics of sarcoptic mange and distemper.

Foxes are suspected to induce strong predation pressure on their prey species (Lindström et al., 1994). The red fox is one of the most widespread generalist mesopredators in Western Europe, preying on small mammals (thus considered as an auxiliary by some farmers e.g. in areas where voles are crop pests) and numerous birds, particularly species nesting on the ground (thus considered a nuisance to poultry by some farmers), and is a major cause of roe deer (*Capreolus capreolus*) fawn mortality (Delcourt et al., 2022) and locally to lambs (White et al., 2000). This makes them a major target of predator control, particularly in areas where game hunting and conservation programmes are applied, e.g. for waterbirds such as curlew (*Numenius arquata*) and northern lapwing (*Vanellus vanellus*) and for mammals like great hamster (*Cricetus cricetus*) in Alsace.

Moreover, the continental increase in the fox population has coincided with the geographical expansion of the parasite *Echinococcus multilocularis* in Western Europe (Combes et al., 2012), its urbanization (Liccioli et al., 2015), and an increase in the incidence of human alveolar echinococcosis (Schweiger et al., 2007; Said-Ali et al., 2013; Deplazes et al., 2017). Evidence has been provided that regulating the circulation of the parasite by the usual means of shooting or trapping foxes is ineffective and even counterproductive (Comte et al., 2017). Other studies suggest that achieving the desired reduction in parasite circulation through sustained suppression of the fox population to very low levels would require measures that are ethically, logistically, and ecologically unacceptable (Raoul et al., 2003; Jiguet, 2020; Giraudoux et al., 2020). Actually, an optimal form of prevention comprises encouraging people not to handle foxes, fence off gardens, and deworm dogs and cats. Additional prophylactic measures can even be applied locally if necessary, such as deworming by baiting foxes that frequent the surroundings of homes (Comte et al., 2013; Craig et al., 2017), with the advantage of not destabilizing the population and therefore not leaving the space empty for new infected arrivals.

In the context of One Health, this example shows how good for one target can turn bad for others (see also e.g. Giraudoux et al. (2024) and Taty et al. (2024) for other case studies). Here, the control of a potential human health threat, in this case fox rabies, has triggered a cascade of consequences for epizootics such as sarcoptic mange and distemper, and a zoonosis, alveolar echinococcosis. In addition, the change in fox population dynamics may have had an impact on species of conservation concern and small game (Giraudoux et al., 2020; Delcourt et al., 2022).

**References**

Combes, B., Comte, S., Raton, V., Raoul, F., Boue, F., Umhang, G., Favier, S., Dunoyer, C., Woronoff, N., Giraudoux, P., 2012. Westward Spread of Echinococcus multilocularis in Foxes, France, 2005-2010. Emerging Infectious Diseases 18, 2059–2062. doi:10.3201/eid1812.120219

Comte, S., Raton, V., Raoul, F., Hegglin, D., Giraudoux, P., Deplazes, P., Favier, S., Gottschek, D., Umhang, G., Boue, F., Combes, B., 2013. Fox baiting against Echinococcus multilocularis: Contrasted achievements among two medium size cities. Preventive Veterinary Medicine 111, 147–155. doi:10.1016/j.prevetmed.2013.03.016

Comte, S., Umhang, G., Raton, V., Raoul, F., Giraudoux, P., Combes, B., Boué, F., 2017. Echinococcus multilocularis management by fox culling: An inappropriate paradigm. Prev Vet Med 147, 178–185. https://doi.org/10.1016/j.prevetmed.2017.09.010

Craig, P.S., Hegglin, D., Lighttowlers, M.W., Torgerson, P.R., Wang, Q., 2017. Chapter two - Echinococcosis: Control and Prevention, in: Thompson, R.C.A., Deplazes, P., Lymbery, A.J. (Eds.), Echinococcus and Echinococcosis, Part B, Advances in Parasitology. Academic Press, pp. 55–158. https://doi.org/10.1016/bs.apar.2016.11.002

Delcourt, J., Brochier, B., Delvaux, D., Vangeluwe, D., Poncin, P., 2022. Fox Vulpes vulpes population trends in Western Europe during and after the eradication of rabies. Mammal Review 52, 343–359. doi: 10.1111/mam.12289

Deplazes, P., Rinaldi, L., Rojas, C.A.A., Torgerson, P.R., Harandi, M.F., Romig, T., Antolova, D., Schurer, J.M., Lahmar, S., Cringoli, G., Magambo, J., Thompson, R.C.A., Jenkins, E.J., 2017. Global Distribution of Alveolar and Cystic Echinococcosis, in: Thompson, R.C.A., Deplazes, P., Lymbery, A.J. (Eds.), Echinococcus and Echinococcosis, Pt A, Advances in Parasitology. pp. 315–493. https://doi.org/10.1016/bs.apar.2016.11.001

Giraudoux, P., Levret, A., Afonso, E., Coeurdassier, M., Couval, G., 2020. Numerical response of predators to large variations of grassland vole abundance and long-term community changes. Ecology and Evolution 10, 14221–14246. doi:10.1002/ece3.7020

Giraudoux, P., Vuitton, D.A., Craig, P.S., 2024. One Health in practice: a critical reflection on the elimination of alveolar echinococcosis in Zhang and Ming counties, Gansu province, China. Bulletin de l’Académie Vétérinaire de France epub, 14–26. https://doi.org/10.3406/bavf.2024.71084

Jiguet, F., 2020. The Fox and the Crow. A need to update pest control strategies. Biological Conservation 248, 108693. doi:10.1016/j.biocon.2020.108693

Liccioli, S., Giraudoux, P., Deplazes, P., Massolo, A., 2015. Wilderness in the ‘city’ revisited: different urbes shape transmission of Echinococcus multilocularis by altering predator and prey communities. Trends in Parasitology 31, 297–305. doi:10.1016/j.pt.2015.04.007

Lindström, E.R., Andren, H., Angelstam, P., Cederlund, G., Hornfeldt, B., Jaderberg, L., Lemnell, P.A., Martinsson, B., Skold, K., Swenson, J.E., 1994. Disease reveals the predator: Sarcoptic mange, red fox predation, and prey populations. Ecology 75, 1042–1049.

Raoul, F., Michelat, D., Ordinaire, M., Decote, Y., Aubert, M., Delattre, P., Deplazes, P., Giraudoux, P., 2003. Echinococcus multilocularis: secondary poisoning of fox population during a vole outbreak reduces environmental contamination in a high endemicity area. International Journal For Parasitology 33, 945–954.

Said-Ali, Z., Grenouillet, F., Knapp, J., Bresson-Hadni, S., Vuitton, D.A., Raoul, F., Richou, C., Millon, L., Giraudoux, P., the FrancEchino Network, 2013. Detecting nested clusters of human alveolar echinococcosis. Parasitology 140, 1693–1700. https://doi.org/10.1017/s0031182013001352

Schweiger, A., Ammann, R.W., Candinas, D., Clavien, P.A., Eckert, J., Gottstein, B., Halkic, N., Muellhaupt, B., Prinz, B.M., Reichen, J., Tarr, P.E., Torgerson, P.R., Deplazes, P., 2007. Human Alveolar Echinococcosis after Fox Population Increase, Switzerland. Emerging Infectious Diseases 13, 878–882. doi:10.3201/eid1306.061074

Taty, N., Bompangue, D., Moore, S., Muyembe, J.J., de Richemond, N.M., 2024. Spatiotemporal dynamics of cholera hotspots in the Democratic Republic of the Congo from 1973 to 2022. BMC Infectious Diseases 24, 360. https://doi.org/10.1186/s12879-024-09164-9

Wandeler, A.I., 2004. Epidemiology of fox rabies in Europe, in: King, A.A., Fooks, A.R., Aubert, M., Wandeler, A.I. (Eds.), Historical Perspective of Rabies in Europe and the Mediterranean Basin. World Organisation for Animal Health (OIE), Paris, France, pp. 201–214.

White, P.C.L., Groves, H.L., Savery, J.R., Conington, J., Hutchings, M.R., 2000. Fox predation as a cause of lamb mortality on hill farms. Veterinary Record 147, 33–37. https://doi.org/10.1136/vr.147.2.33
